# Contribution of Invariant Natural Killer T Cells to the Clearance of *Pseudomonas aeruginosa* from Skin Wounds

**DOI:** 10.3390/ijms22083931

**Published:** 2021-04-10

**Authors:** Hiromasa Tanno, Emi Kanno, Suzuna Sato, Yu Asao, Mizuki Shimono, Shiho Kurosaka, Yukari Oikawa, Shinyo Ishi, Miki Shoji, Ko Sato, Jun Kasamatsu, Tomomitsu Miyasaka, Hideki Yamamoto, Keiko Ishii, Yoshimichi Imai, Masahiro Tachi, Kazuyoshi Kawakami

**Affiliations:** 1 Department of Science of Nursing Practice, Tohoku University Graduate School of Medicine, 2-1 Seiryo-cho, Aoba-ku, Sendai 980-8575, Japan; ekanno@med.tohoku.ac.jp (E.K.); suzuna.s.0321@gmail.com (S.S.); yuuu.sr18723@icloud.com (Y.A.); mizuki.bmty@gmail.com (M.S.); 2 Department of Plastic and Reconstructive Surgery, Tohoku University Graduate School of Medicine, 2-1 Seiryo-cho, Aoba-ku, Sendai 980-8575, Japan; shihoko_5x2@yahoo.co.jp (S.K.); ishishinyoushi@yahoo.co.jp (S.I.); miki_shouji_0121@yahoo.co.jp (M.S.); yo-imai@med.tohoku.ac.jp (Y.I.); tachi@med.tohoku.ac.jp (M.T.); 3 Department of Medical Microbiology, Mycology and Immunology, Tohoku University Graduate School of Medicine, 2-1 Seiryo-cho, Aoba-ku, Sendai 980-8575, Japan; yukari.oikawa.s1@dc.tohoku.ac.jp (Y.O.); ishii-k@med.tohoku.ac.jp (K.I.); kawakami@med.tohoku.ac.jp (K.K.); 4 Department of Intelligent Network for Infection Control, Tohoku University Graduate School of Medicine, 2-1 Seiryo-cho, Aoba-ku, Sendai 980-8575, Japan; ko-sato@med.tohoku.ac.jp (K.S.); kasamatsu@med.tohoku.ac.jp (J.K.); 5 Division of Pathophysiology, Department of Pharmaceutical Sciences, Faculty of Pharmaceutical Sciences, Tohoku Medical and Pharmaceutical University, Sendai 981-8558, Japan; t-miya13@tohoku-mpu.ac.jp; 6 Graduate School of Health Sciences, Niigata University, 2-746 Asahimachi-dori, Chuo-ku, Niigata 951-8518, Japan; hyamamoto@clg.niigata-u.ac.jp

**Keywords:** iNKT cell, skin wound healing, *Pseudomonas aeruginosa*, antimicrobial peptide, IL-17A, IL-22, IFN-γ

## Abstract

Chronic infections are considered one of the most severe problems in skin wounds, and bacteria are present in over 90% of chronic wounds. *Pseudomonas aeruginosa* is frequently isolated from chronic wounds and is thought to be a cause of delayed wound healing. Invariant natural killer T (iNKT) cells, unique lymphocytes with a potent regulatory ability in various inflammatory responses, accelerate the wound healing process. In the present study, we investigated the contribution of iNKT cells in the host defense against *P. aeruginosa* inoculation at the wound sites. We analyzed the re-epithelialization, bacterial load, accumulation of leukocytes, and production of cytokines and antimicrobial peptides. In iNKT cell–deficient (Jα18KO) mice, re-epithelialization was significantly decreased, and the number of live colonies was significantly increased, when compared with those in wild-type (WT) mice on day 7. IL-17A, and IL-22 production was significantly lower in Jα18KO mice than in WT mice on day 5. Furthermore, the administration of α-galactosylceramide (α-GalCer), a specific activator of iNKT cells, led to enhanced host protection, as shown by reduced bacterial load, and to increased production of IL-22, IL-23, and S100A9 compared that of with WT mice. These results suggest that iNKT cells promote *P. aeruginosa* clearance during skin wound healing.

## 1. Introduction

Normal wounds heal within two weeks and consist of inflammatory, proliferative, and remodeling phases. In chronic wounds, such as pressure ulcers and diabetic leg ulcers, however, microbial clearance becomes stagnant, and this leads to persistent inflammation, which causes delayed wound healing. Bacterial infection is one cause of chronic wounds. Bacteria are detected in more than 90% of chronic wounds [1], and *Pseudomonas aeruginosa* is one of the most commonly isolated bacteria from chronic wounds [2,3]. Various cells, including keratinocytes, neutrophils, macrophages, and lymphocytes, contribute to the clearance of *P. aeruginosa* through the production of antimicrobial peptides (AMPs) and cytokines [4,5,6,7,8,9].

Both leukocytes and keratinocytes play an important role in skin wound healing and host defense against bacterial infection. Within the innate immune response, phagocytic cells, such as neutrophils and macrophages, infiltrate the wound sites and eradicate necrotic tissue debris and invading pathogens [10]. In addition, keratinocytes release AMPs, such as S100 proteins, β-defensins, and cathelicidins, which have been reported to promote not only skin wound healing but also the clearance of pathogens [11,12]. Several studies have shown that both phagocytic cell migration and AMP production by keratinocytes is induced by interleukin (IL)-17A and IL-22 [13,14,15,16]. These cytokines are produced by lymphocytes, including T cells, γδT cells, innate lymphoid cells, and natural killer T (NKT) cells [14].

Invariant NKT (iNKT) cells express an invariant TCRα chain (Vα14–Jα18 in mice and Vα24–Jα18 in humans) in combination with a certain TCRβ chain. iNKT cells produce a variety of cytokines, such as interferon (IFN)-γ, IL-4, IL-17A, and IL-22, when they recognize a glycolipid antigen, especially α-galactosylceramide (α-GalCer) presented in context with CD1d, an MHC class I-like molecule [17,18]. Therefore, iNKT cells are thought to orchestrate immune responses through their ability to produce a variety of cytokines. Our previous studies showed that iNKT cells infiltrate injured skin tissues and accelerate wound healing [19,20]. Another group reported that iNKT cell activation by α-GalCer resulted in promoted *P. aeruginosa* clearance from the lung by inducing macrophage activation and IFN-γ production [7]. Thus, iNKT cells have been shown to be involved in the wound healing process and protection from *P. aeruginosa* infection. Their role in the wound healing process under a condition with *P. aeruginosa* infection remains to be elucidated.

Against this background, in the present study, we analyzed the effects of iNKT cell deficiency on the healing process in *P. aeruginosa*–inoculated skin wounds using Jα18 knockout (KO) mice genetically lacking iNKT cells.

## 2. Results

### 2.1. Reduced Elimination of P. aeruginosa and Delayed Re-Epithelialization in Jα18KO Mice

**Figure 1:**
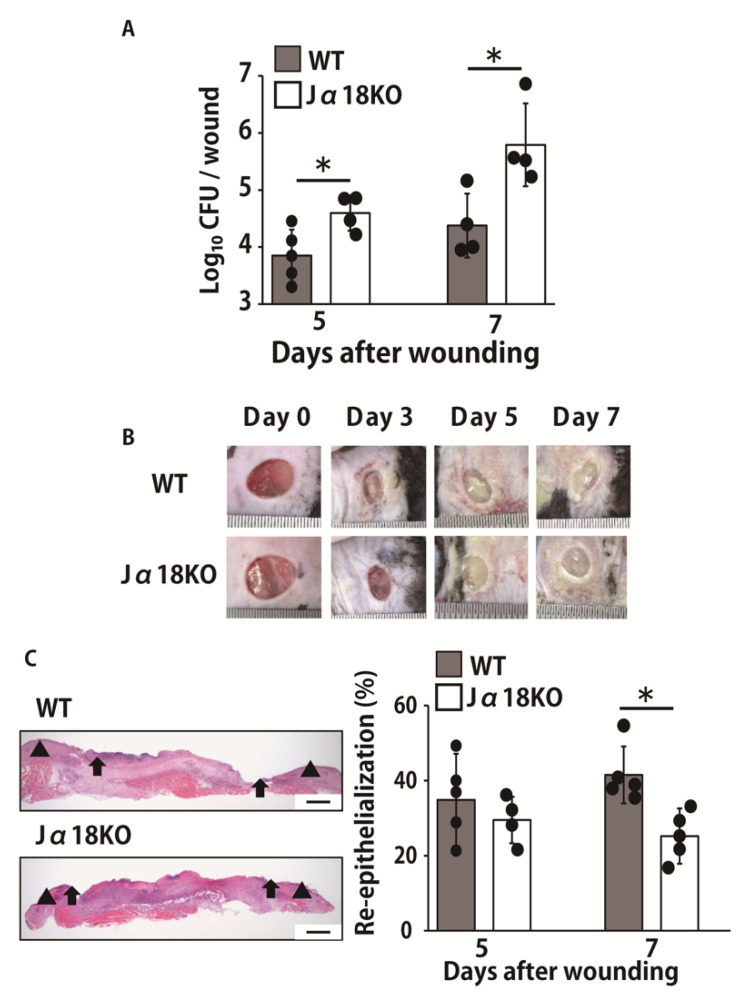
Effect of invariant natural killer T (iNKT) cell deficiency on the clearance of bacteria and re-epithelialization in wounds after inoculation with *P. aeruginosa*. Wounds were created on the backs of WT or Jα18KO mice, and *P. aeruginosa* strain PAO1 (0.7–1 × 10^4^ colony forming units (CFUs)/wound) was directly inoculated at the wound sites immediately after wounding. (**A**) The number of live colonies in the wound on days 5 and 7 postinoculation were counted (n = 4–5). (**B**) Wound photograph in WT or Jα18KO mice. (**C**) Representative histologic views of skin wounds on day 7. Arrowheads and arrows indicate the original wound edges and re-epithelialized leading edges, respectively. Scale bar, 500 μm. Time-course changes in the re-epithelialization ratio after wound creation (n = 4–5). Black circles represent one individual mouse. Each column represents the mean ± standard deviation (SD). The results are representative of at least two independent experiments. * *p* < 0.05.

To clarify the effect of iNKT cell deficiency on the host defense against *P. aeruginosa* infection and skin wound healing, we compared the number of live colonies of *P. aeruginosa* in the wounds between wild-type (WT) and Jα18KO mice. The bacterial burdens in wounds were significantly higher in Jα18KO mice than in WT mice on days 5 and 7 post-infection (Figure 1A). Inoculated wounds showed signs of suppurative inflammation in both mice (Figure 1B). In addition, to examine the possible contribution of iNKT cells to wound healing, the re-epithelialization of wounded skin was compared between Jα18KO mice and WT mice. This response was significantly delayed in Jα18KO mice compared to that in WT mice on day 7 (Figure 1C).

### 2.2. Effect of iNKT Cell Deficiency on Neutrophils and Macrophages Migration at the Wound Sites after Inoculation with P. aeruginosa

**Figure 2:**
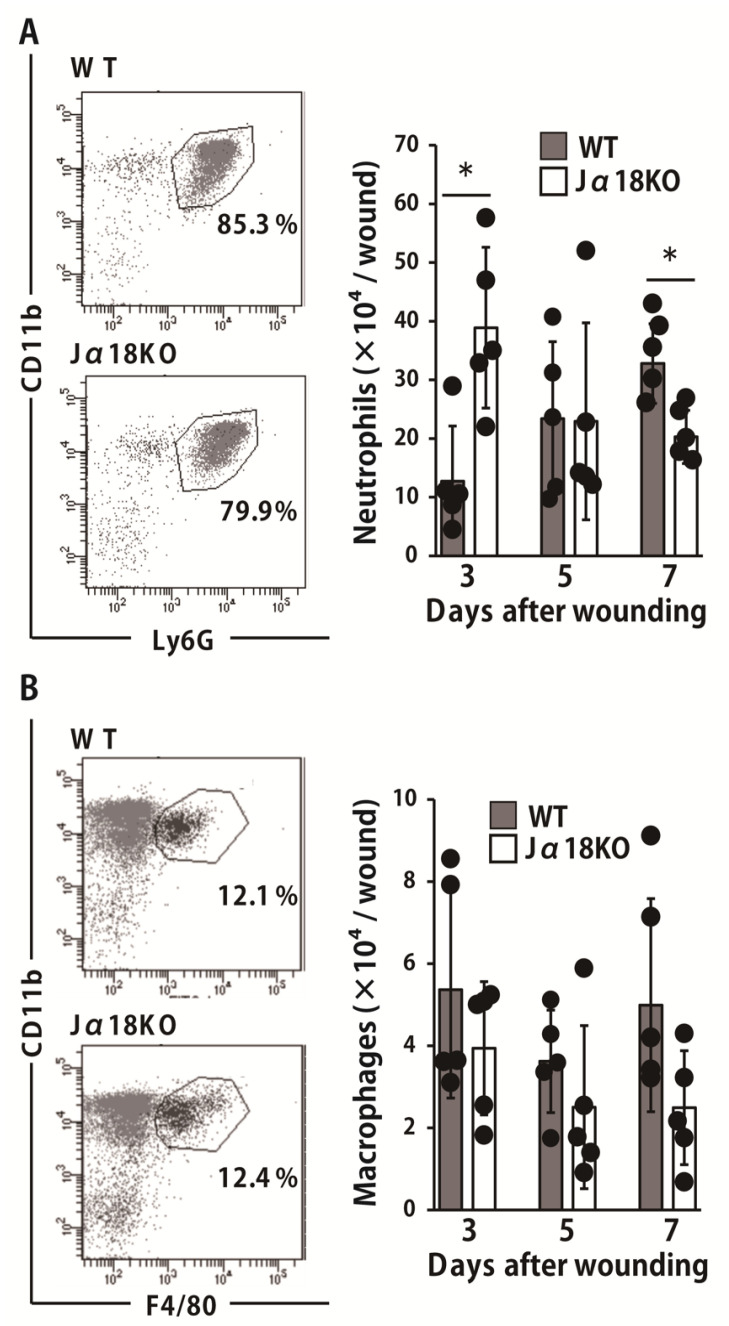
Effect of iNKT cell deficiency on neutrophils and macrophages migration at the wound sites after inoculation with *P. aeruginosa*. Wounds were created on the backs of WT or Jα18KO mice, and *P. aeruginosa* was directly inoculated at the wound sites after wounding. Leukocytes were prepared from wounds at the indicated time points, and the number of neutrophils, defined as CD45^+^Ly6G^+^CD11b^+^ cells (**A**), and the number of macrophages, defined as CD45^+^CD11b^+^F4/80^+^ cells was analyzed (**B**). Representative dot plots on day 7 are shown (**A**,**B**). Black circles represent one individual mouse. Each column represents the means ± SD (*n* = 4–5). The results are representative of at least two independent experiments. * *p* < 0.05.

Neutrophils are innate immune cells that accumulate in the early phase of the wound healing process and play an important role in the eradication of bacteria. To elucidate the role of iNKT cells in the neutrophil-mediated host defense against this bacterium, we evaluated the number of migrated neutrophils in the wounded tissues. As shown in Figure 2A, the neutrophil counts were significantly higher in Jα18KO mice than they were in WT mice on day 3 whereas the opposite results were obtained on day 7 after wound creation. There was no significant difference in the infiltration of macrophages (Figure 2B).

### 2.3. Effect of iNKT Cell Deficiency on the Production of Antimicrobial Peptides at the Wound Sites after Inoculation with P. aeruginosa

**Figure 3:**
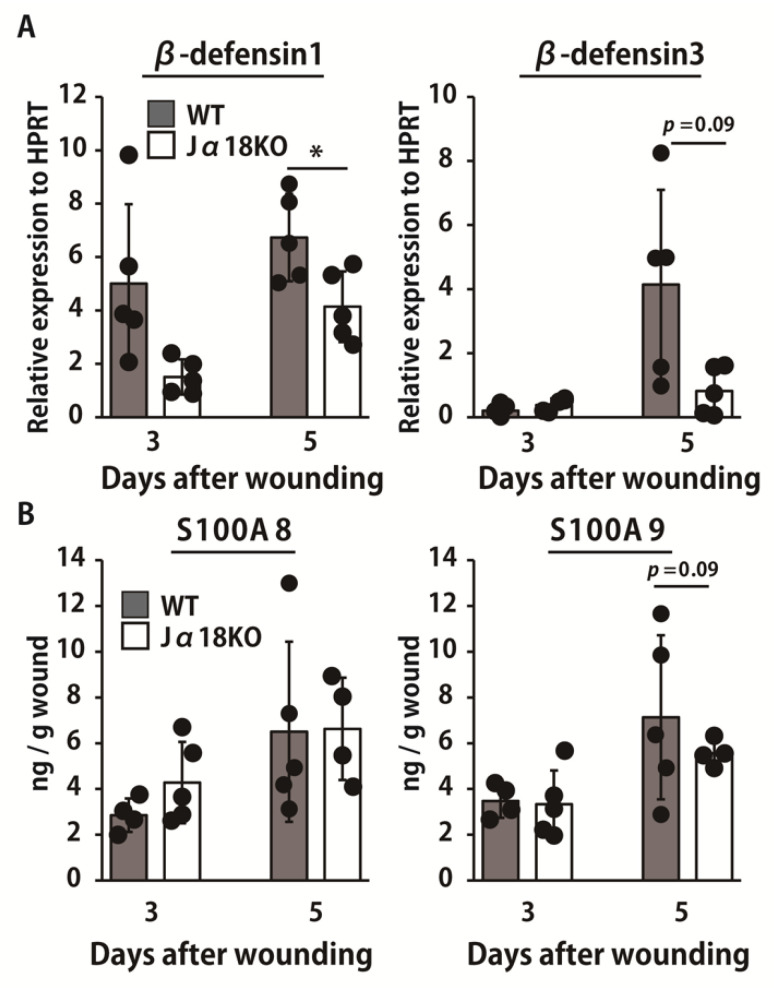
Effect of iNKT cell deficiency on the production of antimicrobial peptides at the wound sites after inoculation with *P. aeruginosa*. WT and Jα18KO mice were wounded and inoculated with *P. aeruginosa*. (**A**) Expression of β-defensin1 and β-defensin3 in the wounded tissues was measured on days 3 and 5 (n = 4–5). (**B**) Production of S100A8 and S100A9 in the wound tissue homogenates were measured at each specified point (n = 4–5). Black circles represent one individual mouse. Each column represents the means ± SD. The results are representative of at least two independent experiments. * *p* < 0.05.

AMPs are known to control bacterial growth as an innate immune system. Therefore, we examined the production of AMPs, such as β-defensin1, β-defensin3, S100A8, and S100A9. As shown in Figure 3A, the expression of β-defensin1 was significantly lower in Jα18KO mice than it was in WT mice on day 5. β-defensin3 expression was decreased in Jα18KO mice compared with that in WT mice, although this difference was not statistically significant. In addition, there was no significant difference in the production of S100A8 and S100A9 between the two groups on days 3 and 5 (Figure 3B).

### 2.4. Effect of iNKT Cell Deficiency on the Production of Cytokines at the Wound Sites after Inoculation with P. aeruginosa

**Figure 4:**
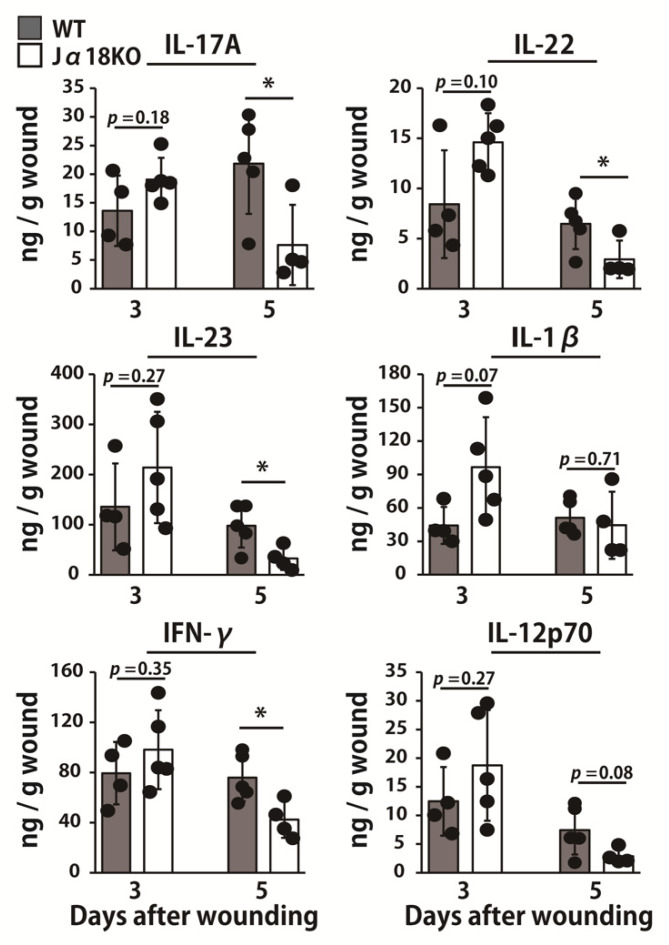
Effect of iNKT cell deficiency on the production of cytokines at the wound sites after inoculation with *P. aeruginosa*. WT and Jα18KO mice were wounded and inoculated with *P. aeruginosa*. IL-17A, IL-22, IL-23, IL-1β, IFN-γ, and IL-12p70 levels in the wounded tissues were measured on days 3 and 5 (n = 4–5). Black circles represent one individual mouse. Each column represents the means ± SD. The results are representative of at least two independent experiments. * *p* < 0.05.

Next, we addressed the possible involvement of iNKT cells in the synthesis of cytokines important for neutrophil-mediated inflammatory responses and AMP production. As shown in Figure 4, the production of IL-17A, IL-22, IL-23, and IFN-γ was significantly decreased in Jα18KO mice compared with that in WT mice on day 5 whereas IL-12p70 was reduced in Jα18KO mice compared to that of WT mice, although this difference was not statistically significant.

### 2.5. Effect of iNKT Cell Activation on the Elimination of P. aeruginosa

**Figure 5:**
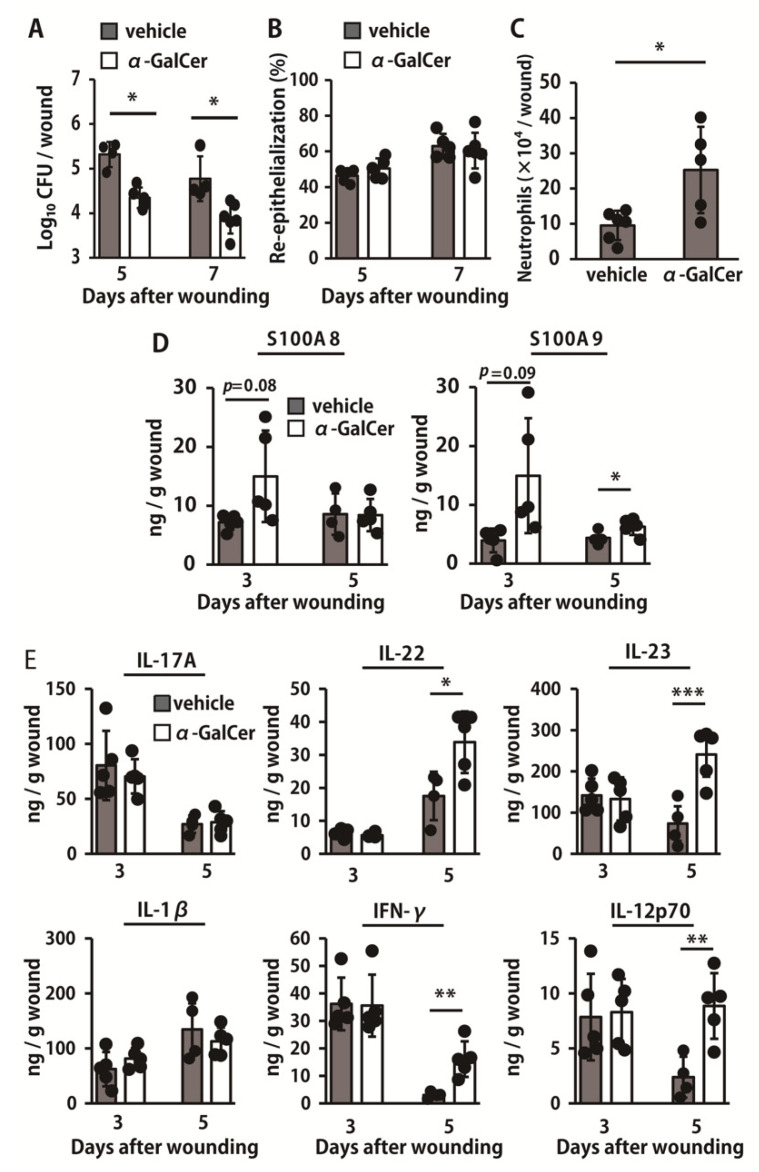
Effect of iNKT cell activation on the bacterial loads, re-epithelialization, neutrophil accumulation, and production of antimicrobial peptides and cytokines after infection with *P. aeruginosa.* Wounds were created on the backs of vehicle- or α-GalCer-treated mice and *P. aeruginosa* strain PAO1 (0.7–1 × 10^4^ CFUs/wound) was directly inoculated at the wound sites immediately after wounding. (**A**) The number of live colonies in the wound on days 5 and 7 postinoculation were counted (n = 4–5). (**B**) Time-course changes in the re-epithelialization ratio after wound creation (n = 5). (**C**) The number of neutrophils, defined as CD45^+^Ly6G^+^CD11b^+^ cells, was analyzed on day 3 (n = 5–6). (**D**) Production of S100A8 and S100A9 in the wound tissue homogenates were measured at each specified point (n = 4–5). (**E**) IL-17A, IL-22, IL-23, IL-1β, IFN-γ, and IL-12p70 levels in the wounded tissues were measured on days 3 and 5 (n = 4–5). Black circles represent one individual mouse. Each column represents the mean ± SD. The results are representative of at least two independent experiments. * *p* < 0.05, ** *p* < 0.01, *** *p* < 0.005.

To further address the possible contribution of iNKT cells, we examined the effects of administration of α-GalCer, a specific activator of these cells, on the clearance of *P. aeruginosa*. α-GalCer treatment significantly reduced the number of live bacteria in the wounds on days 5 and 7, when compared to the vehicle treatment (Figure 5A). However, the ratio of re-epithelialization was not significantly different between the two groups (Figure 5B). The number of neutrophils was significantly higher in α-GalCer-treated mice than that in vehicle-treated mice on day 3 (Figure 5C). As shown in Figure 5D, S100A9 production in α-GalCer-treated mice was significantly increased compared to that in vehicle-treated mice on day 5 whereas S100A8 and S100A9 production tended to be increased in α-GalCer-treated mice compared to that in vehicle-treated mice on day 3. In addition, α-GalCer-treated mice exhibited increased production of IL-22, IL-23, IFN-γ, and IL-12p70 compared with that of vehicle-treated mice on day 5 (Figure 5E).

## 3. Discussion

The role of iNKT cells in pulmonary infection with *P. aeruginosa* has been previously reported [7]. Although we previously reported that iNKT cells infiltrated wound tissues [19], their role in the wound healing process under a condition with *P. aeruginosa* infection remains unclear. In the present study, we demonstrated for the first time that *P. aeruginosa* clearance and re-epithelialization in skin was delayed under a condition lacking iNKT cells and that activation of iNKT cells led to the accelerated elimination of this bacterium. iNKT cells have been reported to play an important role in protection against bacterial infections [21]. Because neutrophils mainly eliminate *P. aeruginosa* by phagocytic killing, the increase in the number of these cells at the infected tissues is thought to be important for bacterial clearance [22,23]. In the current study, neutrophil number was significantly decreased in Jα18KO mice compared to that of WT mice. Similar results were reported in which the number of neutrophils was decreased in CD1dKO mice lacking NKT cells [7]. In contrast, however, we observed an increased number of neutrophils in Jα18KO mice at the early time points after wound creation, although the clearance of *P. aeruginosa* at the wounded tissues was impaired. Benoit et al. showed that CD1dKO mice exhibited promoted recruitment of neutrophils compared to that of WT mice at the early time points, but the elimination of *P. aeruginosa* by these cells was impaired under the condition lacking NKT cells [24]. These observations suggest that iNKT cells may be deeply involved not only in the early phase migration of neutrophils but also in their function of phagocytic killing. Moreover, Wang et al. reported that early production of IL-4 by iNKT cells promoted neutrophil survival, whereas IFN-γ sequentially produced by these cells after IL-4 production induced neutrophil apoptosis in hepatitis model [25], suggesting that iNKT cells may have opposite roles in neutrophilic responses at different phases.

Macrophages are also important immune cells to eradicate invading pathogens, and their killing ability is activated by IFN-γ [26,27]. In previous studies, it was demonstrated that the killing of *P. aeruginosa* by macrophages was enhanced in the presence of IFN-γ [27]. In addition, impaired clearance of this bacterium was observed in IFN-γKO mice [28]. Furthermore, our current data showed that IFN-γ production was reduced in Jα18KO mice compared with that of WT mice. Collectively, these findings suggest that iNKT cells may be deeply involved in the clearance of *P. aeruginosa* in skin through promoting macrophage phagocytic killing by IFN-γ production, although the number of macrophages was not markedly different between WT and Jα18KO mice.

AMPs, such as β-defensins and S100 proteins, have been reported as important molecules in the host defense against microorganisms [29,30]. The expression of AMPs in epithelial cells is up-regulated after infection with *P. aeruginosa* [31,32]. Morrison and colleagues revealed that β-defensin1 exerted antibacterial effects against this bacterium [33]. Furthermore, during the skin wound healing process, AMPs stimulate keratinocyte proliferation, which leads to the early completion of epithelialization [11,12]. In the current study, we observed the decreased expression of β-defensin1 in Jα18KO mice, suggesting that iNKT cells may enhance the bacterial clearance and epithelialization by regulating AMP production.

Two types of signals are involved in the activation of iNKT cells: T cell receptor (TCR)-dependent activation and cytokine-dependent activation [34]. TCR-dependent activation, mediated by the recognition of glycolipid antigens by iNKT cell-TCR, leads to the production of a large number of cytokines, such as IFN-γ, IL-17A, and IL-22 [35]. In contrast, cytokine-dependent activation requires the recognition of pathogen-associated molecular patterns (PAMPs) by pattern recognition receptors (PRRs) expressed on the surface of antigen presenting cells (APCs) [35]. When infected with bacteria, APCs secrete cytokines, including IL-12 and IL-23, that activate iNKT cells in a TCR-independent manner [35]. To the best of our knowledge, no studies have reported any glycolipid antigen of *P. aeruginosa* recognized by iNKT cells [36]. Thus, we believe that iNKT cells may be activated during *P. aeruginosa* infection in a cytokine-dependent, rather than a TCR-dependent, manner. Previous studies showed that APCs secrete IL-1β and IL-23 upon stimulation with lipopolysaccharide (LPS), a component of *P. aeruginosa* [36,37], which may result in the activation of iNKT cells [38]. We found that IL-1β and IL-23 production was not markedly different between WT mice and Jα18KO mice on day 3 after wounding, although IL-17A and IL-22 production was decreased in Jα18KO mice on day 5. These observations suggest that the ability of APCs to produce IL-1β and IL-23 may not differ between WT and Jα18KO mice, and that IL-17A and IL-22 production may be caused by the activated iNKT cells. IL-17A and IL-22 are known to contribute to the epithelial barrier function against bacterial infection through inducing the production of AMPs, including β-defensins and S100 proteins [11,39]. Consistent with the results of previous studies, we found that AMP production was significantly reduced in Jα18KO mice compared with that of WT mice, which was accompanied by the reduced production of IL-17A and IL-22.

In the present study, we also examined the effect of α-GalCer administration on *P. aeruginosa* clearance in the skin. Activation of iNKT cells by α-GalCer led to promoted clearance of *P. aeruginosa*, neutrophil infiltration, and the production of AMPs and cytokines. Earlier studies reported that treatment with α-GalCer accelerated the clearance of *P. aeruginosa* in the lungs, which was associated with the increased production of IFN-γ [7]. Unexpectedly, however, the re-epithelialization was not increased in α-GalCer-treated mice, even though this indicator was significantly attenuated in iNKT cell-deficient mice. Because excessive inflammatory cytokine production was reported to cause delayed healing [40,41,42], stimulation of immune cells with *P. aeruginosa* in the presence of α-GalCer may result in the excessive production of inflammatory cytokines from iNKT cells, which may be involved in the promotion of bacterial clearance and the suppression of re-epithelialization.

In conclusion, we speculate that iNKT cells may be involved in the production of IL-17A, IL-22, and IFN-γ when wounds are infected with *P. aeruginosa*, and these cells may regulate the production of AMPs in the epidermis to accelerate wound repair and the elimination of *P. aeruginosa.* In contemporary practice, many patients suffer from chronic skin wounds, and this bacterium is detected in more than 50% of these wounds [43]. Thus, our findings may suggest a novel therapeutic approach for the treatment of chronic wounds in the skin.

## 4. Materials and Methods

### 4.1. Animals

iNKT cell–deficient mice (Jα18KO mice), established by targeted deletion of the Jα18 gene segment, [44] were kindly provided by Dr. Toshinori Nakayama (Chiba University, Chiba, Japan). This mouse was back-crossed more than eight times with C57BL/6J mice. Wild-type (WT) C57BL/6J mice, purchased from CLEA Japan (Tokyo, Japan), were used as controls. Male or female mice at 7 to 10 weeks of age were used in the experiments. Food and water were available ad libitum. All mice were kept under specific pathogen-free conditions in the Institute for Animal Experimentation, Tohoku University Graduate School of Medicine (Sendai, Japan). Purchased mice were acclimatized for at least a week before wounding. We took the utmost care to alleviate any pain and suffering in mice during the experiments.

### 4.2. Bacteria

*P. aeruginosa* (PAO-1 strain) were prepared as described previously [45]. Briefly, *P. aeruginosa* were inoculated onto a sheep blood agar plate and incubated at 37 °C overnight, and colonies were cultured in brain heart infusion (BHI) broth (Eiken Chemical Co., Ltd., Tokyo, Japan) at 37 °C for 24 h and washed three times in normal saline. After the final suspension was mixed, bacterial counts were performed by measuring the absorbance at 600 nm. In each experiment, a quantification culture was performed to confirm the inoculation dose.

### 4.3. Wound Creation and Tissue Collection

All handling of the animals was performed under anesthesia induced by an intraperitoneal injection of 40 mg/kg sodium pentobarbital (Somnopentyl, Kyoritsu Seiyaku Corporation, Tokyo, Japan) and sustained by inhalation anesthesia of isoflurane (Isoflurane, Mairan Pharma, Osaka, Japan). The dorsal hair was shaved using hair clippers (Model 2100, Daito Electric Machine Industry Co., Ltd., Osaka, Japan) to fully expose the skin, which was then rinsed with 70% ethanol. Two full-thickness wounds extending to the panniculus carnosus were created using a 6-mm-diameter biopsy punch (Biopsy Punch, Kai industries Co., Ltd., Gifu, Japan) under sterile conditions. A suspension (10 μL) of *P. aeruginosa* PAO1 was applied to the base of the wounds at 0.7 to 1.0 × 10^4^ CFUs/wound in an individual mouse. *P. aeruginosa* solution was prepared in every experiment to maintain viability, and it was therefore not possible to prepare them at exactly the same inoculum dose. The injured areas were covered with a polyurethane film (Tegaderm Transparent Dressing, 3M Health Care, St. Paul, MN, USA) and an elastic adhesive bandage (Hilate, Iwatsuki, Tokyo, Japan) as an occlusive dressing. The wounded mice were housed five to six per cage after wound creation, and male and female mice were housed in separate cages. The day on which the wounds were made was designated as day 0. At various time points, mice were sacrificed and the wound tissue was collected by excising a 1-cm-square section of skin using scissors and a surgical knife. Mice were sacrificed by cervical dislocation prior to analysis.

### 4.4. Reagents and Antibodies

α-GalCer was purchased from Funakoshi (Tokyo, Japan) and dissolved in dimethyl sulfoxide (DMSO) at 5 mg/mL, which was diluted with phosphate buffered saline (PBS). The final dose for use in vivo was 0.2% DMSO in PBS. Therefore, PBS containing 0.2% DMSO (dPBS) was used as the control vehicle. To activate iNKT cells, mice were injected intraperitoneally with α-GalCer (2 μg/mouse) on day 1 before wound creation and on day 3 after wound creation.

### 4.5. Counting the Viable P. aeruginosa

Mice were sacrificed on day 5 or 7 after wounding, and the wounded tissues were dissected carefully and excised. They were then homogenized in normal saline by teasing through a stainless mesh at room temperature. The homogenates (100 μL) were diluted in tenfold series using sterile normal saline and inoculated onto a nalidixic acid cetrimide agar plate (Eiken Chemical Co., Ltd., Tokyo, Japan). The homogenate was then cultured for 24 h at 37 °C, and the number of colonies was counted.

### 4.6. Histological Analysis

The tissues were fixed with 4% paraformaldehyde-phosphate buffer solution and embedded in paraffin as previously described [16]. Sections were taken from the central portion of the wound and stained with hematoxylin and eosin (HE) according to the standard method. The extent of re-epithelialization of each wound was measured in these HE-stained sections by measuring the distance from the normal wound margin to the edge of the epithelium. The re-epithelialization index was determined based on the percentage of new epithelium present in the total wound.

### 4.7. RNA Extraction and Quantitative Real-Time Reverse Transcription Polymerase Chain Reaction (RT-PCR)

Total RNA was extracted from the wound tissues using ISOGEN (Nippon Gene Co. Ltd., Tokyo, Japan), and first-strand cDNA was synthesized using a PrimeScript first-strand cDNA synthesis kit (TaKaRa Bio Inc., Otsu, Japan) according to the manufacturer’s instructions. Quantitative real-time polymerase chain reaction (PCR) was performed in a volume of 20 μL using gene-specific primers and FastStart essential DNA green master mix (Roche Applied Science, Penzburg, Germany) in a Step One^TM^ (Thermo Fisher Scientific, Waltham, MA, USA). Primers were as follows: 5′- CGT TGG GCT TAC CTC ACT GC -3′ (Forward) and 5′- ATC GCT AAT CAC GAC GCT GG -3′ (Reverse) for HPRT; 5′-CTT TTC TCC CAG ATG GAG CCA G -3′ (Forward) and 5′- CCT CCA TGT TGA AGG CAT TTG TAT TG -3′ (Reverse) for β-defensin 1; and 5′ -TTC TCC TGG TGC TGC TGT CTC-3′ (Forward) and 5′-GCC TCC TTT CCT CAA ACA ACT TA-3′ (Reverse) for β-defensin3. The reaction efficiency with each primer set was determined using standard amplifications. Target gene expression levels and that of HPRT as a reference gene were calculated for each sample using the reaction efficiency. The results were analyzed using a relative quantification procedure and are presented as expression levels relative to that of HPRT.

### 4.8. Preparation of Leukocytes in the Wounded Tissue

Leukocytes were prepared as previously described [46]. Briefly, mice were sacrificed on days 3, 5, or 7 after wounding. The wound tissues were excised in a 1 cm square using scissors and a surgical knife and teased apart using stainless-steel mesh in RPMI 1640 medium (Sigma-Aldrich, St. Louis, MA, USA) supplemented with 10 mM HEPES, 10% fetal calf serum (FCS) (BioWest, Nuaillé, France), 1 mg/mL collagenase, and 1 mg/mL hyaluronidase (Sigma-Aldrich). Tissues were then incubated for 2 h at 37 °C with vigorous shaking. After incubation, the tissue fragments and most dead cells were removed when passed through a 70 μm cell strainer (BD Falcon, Bedford, MA, USA). After centrifugation, the cell pellet was resuspended in 4 mL of 40% (*v/v*) Percoll (Cytiva, Tokyo, Japan) and layered onto 4 mL of 80% (*v/v*) Percoll. After centrifugation at 600× *g* for 20 min at 15 °C, the cells at the interface were collected, washed three times, and counted using a hemocytometer.

### 4.9. Flow Cytometry

The cells obtained from the wounded tissues were incubated with Anti-Mouse CD16/CD32 (clone 2.4G2, BD Biosciences, Franklin Lakes, NJ, USA) on ice for 15 min in PBS that contained 1% FCS and 0.1% sodium azide. The cells were stained with Pacific blue-anti-CD45 monoclonal antibody (mAb) (clone 30-F11, BioLegend, San Diego, CA, USA), APC-anti-CD11b mAb (clone M1/70, BioLegend, San Diego, CA, USA), APC/Cy7-anti-Ly6G mAb (clone 1A8, BioLegend, San Diego, CA, USA), and FITC-anti-F4/80 mAb (clone BM8, BioLegend, San Diego, CA, USA). Isotype-matched irrelevant IgG (Pacific: blue Rat IgG2b, κ Isotype Ctrl mAb, APC Rat IgG2b, κ Isotype Ctrl, APC/Cy7 Rat IgG2a, κ Isotype Ctrl, and FITC Rat IgG2a, κ Isotype Ctrl, BioLegend, San Diego, CA, USA) was used for control staining. The stained cells were analyzed using a BD FACS CantoTM II flow cytometer (BD Biosciences, Franklin Lakes, NJ, USA) and analyzed using FACS Diva software. Neutrophils and macrophages were identified as CD45^+^CD11b^+^Ly6G^+^ cells and CD45^+^CD11b^+^F4/80^+^ cells, respectively. The number of neutrophils and macrophages was estimated by multiplying the total leukocyte number by the proportion of each fraction.

### 4.10. Measurement of Cytokine Concentrations

The wound tissues were homogenized by teasing through a stainless-steel mesh in saline solution, and the concentration of cytokines and antimicrobial peptides in the supernatants was measured using appropriate enzyme-linked immunosorbent assay (ELISA) kits (BioLegend, San Diego, CA, USA, for IL-17A, IL-22, IL-23p19, IFN-γ, IL-12p70, and IL-1β; R&D Systems, Minneapolis, MI, USA, for S100A8 and S100A9). The results were expressed as the values per wound.

### 4.11. Statistical Analysis

Data were analyzed using JMP^®^ Pro 15 0.0 software (SAS Institute Japan, Tokyo, Japan). Data are expressed as mean ± SD. Differences between groups were examined for statistical significance using Welch’s *t*-test. A *p* value less than 0.05 was considered significant.

## Abbreviations

iNKT cell	invariant natural killer T cell
IL	interleukin
IFN	interferon
AMP	antimicrobial peptide
KO	knockout
WT	wild type
